# Mortality and Health Outcomes for Older Adults Screened by an Area Agency on Aging Over a 4.5-Year Period

**DOI:** 10.1093/geroni/igaa057.2500

**Published:** 2020-12-16

**Authors:** Amber Gum, Lawrence Schonfeld, Kevin Kip, Mary Goldsworthy, Jesse Bell, Kyaien Conner, Ohad Green, Katie Parkinson

**Affiliations:** 1 University of South Florida, Tampa, Florida, United States; 2 University of south florida, Tampa, Florida, United States; 3 University of Oxford, Oxford, England, United Kingdom; 4 Senior Connection Center, Inc., Tampa, Florida, United States

## Abstract

Area Agencies on Aging (AAA) screen older adults and oversee delivery of a wide range of home- and community-based services (HCBS). We examined the assessment process, services, and mortality and health outcomes for older adults screened by an Area Agency on Aging in west-central Florida. Most were self/family referred (78.9%). Using data from July 2013-December 2018, 23,225 older adults were screened. Individuals had an average of 2.6 years follow-up in the dataset, during which time 63.6% received additional assessments: follow-up screening (50.6%), comprehensive assessment for enrollment in HCBS (35.7%), or assessments for congregate meals or other services (13.7%). Results revealed differences in mortality: 22.5% of clients receiving services died compared to 32.1% of clients prioritized as lower risk and on waiting lists for services. Long-term care placement and functional decline outcomes also will be reviewed, along with implications for service delivery and managing waitlists.

